# Early and long-term outcomes of coronary artery bypass surgery with and without use of heart-lung machine and with special respect to renal function - A retrospective study

**DOI:** 10.1371/journal.pone.0223806

**Published:** 2019-10-10

**Authors:** Julia Merkle, Jaison Sunny, Laura Ehlscheid, Anton Sabashnikov, Carolyn Weber, Kaveh Eghbalzadeh, Ilija Djordjevic, Oliver Liakopoulos, Yeong-Hoon Choi, Thorsten Wahlers, Mohamed Zeriouh

**Affiliations:** Department of Cardiothoracic Surgery, University Hospital of Cologne, Cologne, Germany; Azienda Ospedaliero Universitaria Careggi, ITALY

## Abstract

The aim of our study was to compare early and long-term outcome of patients undergoing either on-pump or off-pump coronary artery bypass grafting with special focus on impairment of renal function. Five hundred ninety-three consecutive patients undergoing coronary artery bypass grafting were retrospectively analyzed. They were assigned either to on-pump (n = 281) or to off-pump (n = 312) group. Early and long-term outcomes were analyzed with special focus on renal function. Basic demographics and preoperative characteristics did not differ between groups (p>0.05) as well as postoperative renal parameters (p>0.05). Postoperative odds ratios (OR) of off-pump group in comparison to on-pump group were higher without reaching significance in terms of incidence of gastrointestinal complications and pneumonia (OR = 2.23 and 1.61, respectively) as well as hazard ratios (HR) on long-term follow-up for mortality and incidence of myocardial infarction (HR = 1.50 and 2.29, respectively). Kaplan-Meier estimation analysis also revealed similar results for both groups in terms of mid- and long-term survival (Breslow p = 0.062 and Log-Rank p = 0.064, respectively) and for incidence of myocardial infarction (Breslow p = 0.102 and Log-Rank p = 0.103, respectively). Our study suggests that use or not use of coronary artery bypass did not influence postoperative renal function. Odds of early outcomes were similar in both groups as well as incidence of myocardial infarction and mortality in long-term follow-up.

## Introduction

Cardiac surgery with coronary artery bypass grafting is one of the most frequently applied surgeries world-wide. Numerous studies have been performed in order to compare outcomes of coronary artery bypass grafting (CABG) without (OPCAB) and with use of heart-lung machine (HLM) [1–4]. The conflicting results remain a source of debate. Some studies reported favourable outcomes associated with off-pump surgery [4–9].

Large-scale prospective randomized trials have shown trends towards reduced early risks after off-pump surgery. However these early benefits could not be confirmed in terms of long-term follow-up [3, 10, 11]. Also, worse outcomes of off-pump in comparison to on-pump CABG surgery have been reported concerning graft patency as well as for greater need for coronary reintervention up to one year after surgery [12].

Impairment of renal failure is known to be an important risk factor for increased postoperative mortality of patients after on-pump CABG [13–15]. On-pump surgery may be associated with accelerated growth of atherosclerosis plaques and cardiac calcification which increases operative risk. Becoming an established and feasible technique, OPCAB studies provided a greater benefit in patients with risk for development of renal failure after surgery [16, 17]. It is also known that renal failure can lead to advanced atherosclerotic burden due to changes in calcium metabolism. OPCAB may be a better alternative because of decreased blood loss, and shorter hospital stay [1, 18]. Previously published studies comparing the effect of off-pump versus on-pump CABG on clinical end-points with respect to renal impairment have yielded conflicting results [9, 19, 20].

Therefore, to shed further light on this issue, we performed a retrospective analysis to compare early and long-term outcomes of patients undergoing either on-pump or off-pump coronary artery revascularization with special focus on renal impairment.

## Materials and methods

Patients (n = 593) with coronary artery disease were scheduled to undergo coronary artery bypass grafting from January 2009 to October 2012 in our institution and were retrospectively analyzed. Exclusion criteria were as follows: associated valve surgery procedures, supra-aortic vessel diseases and contraindications to aortic cross-clamping. Decisions about the type of treatment were taken according to local practices and patients were assigned to off-pump or to on-pump surgery group. Three hundred and twelve patients received off-pump and 281 patients underwent on-pump surgery.

This study has been carried out in accordance with the Declaration of Helsinki. The study design was a retrospective review of collected registry data. The Ethics Committee waived both the need for ethics approval and informed consent from patients.CABG technique has been previously described [21]. Off-pump surgery was defined as coronary artery bypass grafting (CABG) without use of heart-lung machine (OPCAB) and on-pump surgery was defined as CABG with use of heart-lung machine.

### Statistical analysis

Statistic was performed using Student’s *t*-Test or Mann-Whitney-*U* Test depending if continuous variables are normally distributed or not and Chi-Square Test was applied for categorical variables. Fisher’s exact Test was performed when the minimum expected count of cells was <5. Univariate regression analysis [Odds ratio (OR)] and Cox regression analysis [Hazard Ratio (HR)] were performed to address predictors for early and long-term outcomes. Kaplan-Meier survival estimation analysis was used to address mid- and long-term survival. A *p-*value <0.05 was considered significant. All statistical analysis was performed using SPSS Version 25.0 (IBM Corp, Chicago, IL, USA).

## Results

Basic demographics and preoperative variables did not significantly differ between both groups (p>0.05) (Table 1). Median age at surgery was 69 years in OPCAB group versus 68 years in on-pump group (p = 0.584). Also, gender, weight and urgency of surgery revealed similar results for both groups (p>0.05). In terms of number of comorbidities, incidence of diabetes, hyperlipidemia, arterial hypertension also similar results were found for both groups (p>0.05). Further, incidence of preoperative apoplexy and carotid artery stenosis did not reveal significant differences.

**Table 1:** Demographic and preoperative variables of patients undergoing coronary artery bypass surgery.

	OPCAB	HLM	p-value
Age	69 (58.25;74)	68 (59;74)	0.584
BMI	27 (25;30)	28 (25;30)	0.584
Female	84 (26.9%)	70 (24.9%)	0.577
LVEF (%)	67 (54;70)	59.5 (46;70)	**<0.001**
Vessel CHD	2 (2;3)	3 (3;3)	**<0.001**
Left main stenosis	60 (19.3%)	80 (28.5%)	**0.009**
Number of bypasses			**<0.001**
1	101 (32.4%)	5 (1.8%)	
2	131 (42.0%)	54 (19.2%)	
3	76 (24.4%)	137 (48.8%)	
4	4 (1.3%)	69 (24.6%)	
5	0 (0.0%)	16 (5.7%)	
Urgency of surgery			0.209
Emergency	33 (10.6%)	34 (12.3%)	
Urgent	36 (11.5%)	44 (15.9%)	
Elective	243 (77.9%)	199 (71.8%)	
Comorbidities			
Diabetes mellitus	102 (32.7%)	100 (35.6%)	0.389
Hyperlipidemia	217 (68.6%)	205 (73.0%)	0.361
Arterial Hypertension	296 (94.9%)	269 (95.7%)	0.623
COPD	33 (10.6%)	32 (11.4%)	0.763
PAOD	45 (14.4%)	40 (14.2%)	0.948
Apoplex	21 (6.7%)	19 (6.8%)	0.988
Renal insufficiency	48 (15.4%)	34 (12.1%)	0.247
IABP preoperative	1 (0.3%)	3 (0.7%)	1.000
Carotid artery stenosis	33 (10.6%)	38 (13.5%)	0.270
Rhythm			0.977
Sinus rhythm	280 (90.3%)	254 (90.4%)	
Atrial fibrillation	30 (9.7%)	27 (9.6%)	
Smoker	73 (23.4%)	76 (27.0%)	0.371
Laboratory parameters			
Haemoglobin (g/dL)	13.8 (12.2;14.7)	13.8 (12.7;14.9)	0.232
Hematocrit (%)	40 (37;43)	41 (38;43)	0.307
Creatinine (mg/dL)	0.92 (0.78;1.08)	0.94 (0.79;1.08)	0.897
Urea (mg/dL)	38 (31;47.25)	38 (31;46.25)	0.893
Creatine kinase- MB (U/L)	15 (11;20)	14 (11;20)	0.739
Thrombocytes x 10^9^/L	229 (191;276)	243 (199;283)	**0.027**
Quick (%)	99 (91;106)	99 (89;105)	0.269
aPTT (sec)	26 (24;28)	26 (24;28)	0.354
**Medication**			
Digitalis	4 (1.3%)	3 (1.1%)	1.000
ACE- inhibitors	190 (60.9%)	169 (60.1%)	0.851
Beta- blockers	235 (75.3%)	211 (75.1%)	0.948
Calcium channel blocker	66 (21.3%)	49 (17.9)	0.301
Diuretics	109 (35.2%)	97 (35.4%)	0.952
Statins	229 (73.9%)	187 (68.2%)	0.189
Antidiabetics	94 (30.3%)	68 (24.8%)	0.067
Aspirin	238 (76.8%)	234 (85.1%)	**0.011**

BMI, Body mass index; LVEF, left ventricular ejection fraction; CHD, coronary heart disease; CABG, coronary artery bypass graft; COPD, chronic obstructive pulmonary disease; PAOD, peripheral artery occlusion disease; IABP, intraaortic balloon pump; creatinine kinase MB, creatine kinase muscle brain; aPTT, activated thromboplastin time.

Number of bypasses significantly differed between groups (p<0.001). Off-pump surgery group mostly comprised integration of one or two bypass grafts, whereas on-pump surgery group comprised three to five grafts.

Laboratory parameters were similarly distributed between both groups, such as hemoglobin (p = 0.232) and anticoagulation parameters (p = 0.269). Median of preoperative creatinine value was 0.92 (mg/dL) in OPCAB group versus 0.94 (mg/dL) in on-pump group revealing no statistical significance (p = 0.897). Also, urea did not preoperatively differ between groups (p = 0.893).

Intra- and postoperative variables of patients undergoing coronary artery bypass grafting with and without use of heart-lung machine are presented in Table 2.

**Table 2:** Intraoperative and early postoperative variables of patients undergoing coronary artery bypass surgery.

	OPCAB	HLM	p-value
Cross-clamp time(min)	0	43 (34;51.5)	**<0.001**
Duration of surgery (min)	130 (109;165)	180 (157;210)	**<0.001**
Cardiopulmonary bypass time (min)	0	79 (64;94.5)	**<0.001**
IABP intraoperative	6 (1.9%)	13 (14.7%)	0.057
IABP perioperative	12 (3.9%)	14 (5.1%)	0.464
Catecholamines			0.251
Catecholamines <24h	249 (80.0%)	205 (75.4%)	
Catecholamines 24h- 48h	28 (9.1%)	29 (10.7%)	
Catecholamines >48h	31(10.1%)	38 (14.0%)	
ICU stay (days)	2 (1;3)	2 (1;4)	0.050
Time for intubation (hours)	14 (11;19)	16 (12;21)	**0.002**
Drainage output over 24h (ml)	700 (520;1010)	750 (530;1135)	0.082
IABP postoperative	6 (1.9%)	1 (0.4%)	0.081
Re-thoracotomy	17 (5.4%)	13 (4.7%)	0.685
Gastrointestinal complications	5 (1.6%)	2 (0.7%)	0.457
Infection	18 (5.8%)	18 (6.5%)	0.704
Pneumonia	9 (2.9%)	5 (1.8%)	0.394
Dialysis postoperative	13 (4.2%)	6 (2.2%)	0.178
In- hospital stay (days)	12 (10;14)	13 (11;16)	**<0.001**
**Follow-up (7-year)**			
Re-thoracotomy	0 (0%)	4 (1.4%)	**0.038**
Apoplexy	8 (2.6%)	10 (3.6%)	0.327
Myocardial infarction	14 (4.5%)	5 (1.8%)	0.085
Mortality	60 (19.2%)	31 (11.0%)	**0.044**
**Laboratory parameters**			
Creatinine 1. Day (mg/dL)	1.0 (0.83;1.22)	1.02 (0.83;1.27)	0.505
Creatinine 3. Day (mg/dL)	1.04 (0.86;1.5)	1.06 (0.81;1.57)	0.916
Urea 1. Day (mg/dL)	38 (30;48)	38 (30;49)	0.809
Urea 3. Day (mg(dL)	49 (37;72.25)	54 (42;72)	0.160
Creatine kinase- MB 1. Day (U/L)	18 (14;29)	30 (24;41)	**<0.001**
Creatine kinase- MB 3. Day (U/L)	16 (12;22)	17 (13;24)	0.131
Lactate 1. Day	1.7 (1.3;2.6)	2.1 (1.5;3)	**<0.001**
Lactate 3. Day	1.5 (1.1;2.0)	1.8 (1.3;2.28)	**0.016**

IABP, intra-aortic balloon pump; ICU, intensive care unit.

Cross-clamp and cardiopulmonary bypass time significantly differed between groups (p<0.001)—per definitionem—as expected due to categorization. Duration of surgery revealed significant differences between the groups (p<0.001).

ICU stay was similarly distributed between both groups as well as need for catecholamines (p = 0.251). In terms of development of complications, such as incidence of gastrointestinal complications (p = 0.478) or incidence of pneumonia (p = 0.394) no significant differences could be detected. Patients in OPCAB group stayed 12 days in hospital and patients in on-pump group 13 days.

Survival of 7-year long-term follow-up was similar between both groups (p = 0.061). Incidence of development of postoperative apoplexy and myocardial infarction was similarly distributed between the groups (p = 0.323 and p = 0.137, respectively).

In terms of postoperative laboratory renal function parameters creatinine values did not significantly differ on postoperative day 1 and day 3 (p = 0.505 and p = 0.916). Also, no significant differences were found for urea values between the groups (p = 0.809).

Odds ratios (OR) for early outcomes and hazard ratios (HR) for long-term outcomes are depicted in Table 3. Patients in OPCAB group revealed higher odds for gastrointestinal complications and pneumonia in comparison to on-pump surgery group without reaching significance (OR = 2.23 and 1.61; both p>0.05). During long-term follow-up incidence for myocardial infarction was 2.3-fold and for mortality 1.5-fold higher in OPCAB group than in on-pump group (HR = 2.29 and 1.50, respectively; both p>0.05), whereas vice versa incidence of apoplexy was 37% reduced (HR = 0.63, p>0.05). All variables did not reach significance.

**Table 3:** Odds ratios (OR) and hazard ratios (HR), 95% confidence intervals (95%-CI) and p-values of selected outcome variables of patients undergoing coronary artery bypass surgery.

	OR	95%-CI	p-value
Re-thoracotomy	1.17	0.56–2.45	0.685
Gastrointestinal complications	2.23	0.43–11.60	0.340
Infection	0.88	0.45–1.72	0.704
Pneumonia	1.61	0.53–4.86	0.399
Dialysis postoperative	1.94	0.73–5.18	0.185
	HR	95%-CI	p-value
Re-thoracotomy	0.01	0.00–40.44	0.285
Apoplexy	0.63	0.25–1.60	0.329
Myocardial infarction	2.29	0.82–6.34	0.113
Mortality	1.50	0.98–2.38	0.066

OR and HR in favour of OPCAB in comparison to HLM.

**Fig 1:**
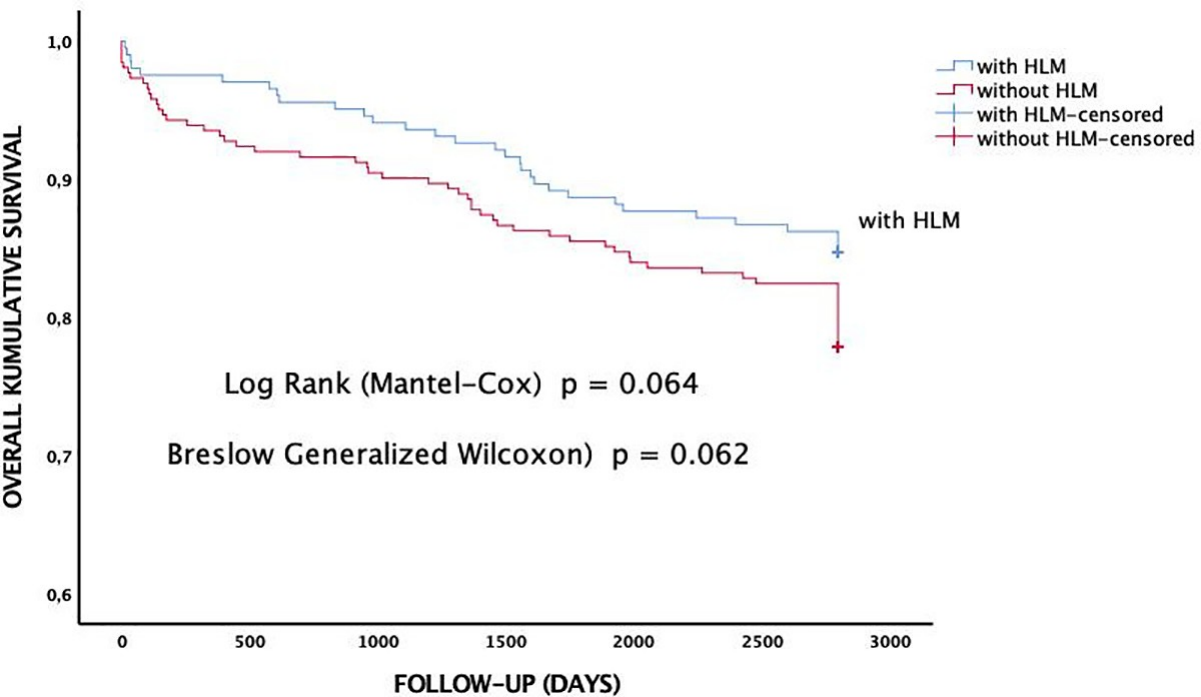
Kaplan-Meier survival estimation plot of coronary artery bypass graft surgery with and without use of heart-lung machine (HLM).

Kaplan-Meier survival estimation analysis depicts mid- (Breslow) and long-term (Log-Rank) survival for up to 7-year follow-up of patients requiring coronary artery bypass surgery with and without use of heart-lung machine revealing similar results between groups (Breslow p = 0.062; Log-Rank p = 0.064) (Fig 1) as well as for incidence of myocardial infarction (Table 4).

**Table 4:** p-values of Kaplan-Meier mid-term (Breslow) and long-term (Log-Rank) estimation analysis up to 7-year follow-up.

Kaplan-Meier		Breslow	Log-Rank
Survival	a	0.062	0.064
Re-thoracotomy	b	0.199	0.197
Myocardial infarction	a	0.102	0.103
Apoplexy	b	0.321	0.324

a in favour of HLM; b in favour of OPCAB.

## Discussion

Coronary artery bypass grafting is one of the most often performed surgeries world-wide with an increasing demand. Many studies revealed that use of heart-lung machine provokes inflammation leading to organ dysfunction and increased mortality [4, 22, 23].

Cardiac surgery with use of cardiopulmonary bypass support initiates a systemic inflammatory response presumably caused by contact of blood components with the artificial surface of the extracorporeal circuit, associated with postoperative morbidity and mortality [22, 24, 25]. In this regard, many studies demonstrated increased inflammatory markers, such as TNF-α, IL-6, IL-8 after cardiac surgery with cardiopulmonary bypass (CPB) inducing organ failure [26]. Massive activation of leukocytes, e.g. neutrophils, and different biochemical pathways may result in microthrombosis, microemboli and depletion of coagulation factors [22]. These factors contribute to tissue injury and endothelial dysfunction, to predisposing patients to organ injury and to increasing perivascular edema [22, 27]. Renal dysfunction can be a common complication after coronary artery bypass surgery with use of heart-lung machine [4, 9]. The inflammatory reaction is induced by use of the heart-lung machine. Some studies revealed a correlation in terms of duration of heart-lung machine with kidney injury [22]. One aim of this study was to evaluate early outcomes with respect to renal function comparing OPCAB with on-pump surgery. Studies revealed controversial results in this field [1, 28, 29]. It has been speculated that coronary artery bypass surgery without use of extracorporeal circulation might positively influence incidence and seriousness of acute renal failure [30]. Li et al. described an incidence of AKI of 37.1% after off-pump surgery [31]. Our study reported similar rates of occurrence of renal failure between groups, confirming the findings of other trials showing that off-pump surgery is not associated with decreased rates or reduced severity of acute renal failure [10, 11]. We also found that impairment of renal function did not differ between groups. Singh et al. measured creatinine up to 4 days postoperatively and the results were similar compared with preoperative levels. They found no statistically significant rise in creatinine in either of the two groups corroborating our results [32]. Thus, they concluded that renal function is not affected by the technique of coronary artery bypass surgery whether with or without cardiopulmonary bypass in spite of the theoretical expected advantage of off-pump surgery [32].

Medved et al. also found no difference in postoperative creatine kinase MB after first day and stay on ICU corroborating our results [20]. Additionally, they revealed similar results in early mortality, whereas we found of a 1.5-fold higher risk for OPCAB group in long-term follow-up (HR = 1.50; p = 0.066) without reaching significance between groups. Also Kaplan-Meier estimation analysis showed similar results between groups (Log-Rank p = 0.064). In our study the number of grafts was similar to the results by Lycops et al. [19]. Interestingly, in terms of 5-year survival Lycops et al. revealed 78% in OPCAB group and 68% in on-pump group, whereas we found a 7-year follow-up survival rate of 81% in OPCAB surgery and 89% in on-pump group. Also, Nicolini et al. found out that on-pump surgery is associated with a lower 5-year mortality than OPCAB group corroborating our results [4]. They also found no differences in terms of incidence of myocardial infarction or stroke affirming our findings. Additionally preoperative statin use does not influence development of postoperative AKI [33].

## Conclusion

Regarding postoperative renal impairment we did not find any differences between patients’ groups undergoing off- or on-pump surgery. Also, odds of early outcomes were similar in both groups as well as incidence of myocardial infarction and mortality in long-term follow-up.

## Study limitations

This study is limited by its retrospective nature, heterogeneity of patient population and relatively small number of patients from a single hospital center. Also, the retrospective nature of this study may introduce confounders and bias of patient selection.

## Supporting information

S1 Data Data set of variables of patients undergoing coronary artery bypass surgery with and without use of heart-lung machine. (SAV)
